# Case Report: Epilepsy-related pure word deafness in the elderly: an underreported presentation?

**DOI:** 10.3389/fresc.2026.1776545

**Published:** 2026-03-20

**Authors:** Toshiaki Takehara, Koji Hayashi, Mamiko Sato, Masumi Yamashita, Hiroaki Maeda, Asuka Suzuki, Yuka Nakaya, Toyoaki Miura, Naoko Takaku, Yasutaka Kobayashi

**Affiliations:** 1 Department of Rehabilitation Medicine, Fukui General Hospital, Fukui, Japan; 2 Graduate School of Health Science, Fukui Health Science University, Fukui, Japan; 3 Department of Neurology, University of Fukui Hospital, Eiheiji-cho, Yo-shida-gun, Fukui, Japan

**Keywords:** case report, dementia, elderly, late-onset epilepsy, pure word deafness, seizure, verbal auditory agnosia

## Abstract

We describe a rare case of epilepsy-related PWD in the elderly. A 61-year-old right-handed man presented with status epilepticus. Although electroencephalogram revealed no epileptiform discharges, brain magnetic resonance imaging (MRI) showed hyperintensity in the left temporoparietal lobe and thalamic pulvinar on diffusion-weighted imaging (DWI). Cerebrospinal fluid revealed no pleocytosis and negative PCR results for herpes simplex virus and varicella-zoster virus. Following the recovery of consciousness, he exhibited a profound dissociation between auditory and speech/language modalities: he was unable to comprehend or repeat spoken language but could follow complex written instructions and fluently produce grammatically correct speech. He had largely preserved perception of non-verbal sounds. Despite minor accompanying oral reading deficits interpreted as concomitant symptoms of an extensive lesion, he was diagnosed with epilepsy-related PWD. Following the administration of levetiracetam, the patient's language function recovered significantly within 20 days. This case suggests that PWD represents an overlooked, transient manifestation of dementia-like symptoms in late-onset epilepsy. Due to its rapid reversibility, such cases are likely underrecognized and underreported; further documentation is warranted.

## Introduction

Pure word deafness (PWD), alternatively referred to as verbal auditory agnosia (AVA), represents an uncommon central auditory condition defined by a specific deficit in understanding spoken language, alongside intact speech production, reading, and writing abilities, as well as largely preserved perception of non-verbal sounds and music (1–8). As a form of auditory agnosia, this condition is attributed to cortical or subcortical dysfunction and is independent of peripheral hearing impairment (9–11). In clinical settings, affected individuals often appear unresponsive to speech as if they were totally deaf, even though standard audiometric assessments are frequently normal or insufficient to explain the degree of impairment (1, 2, 5, 6, 8). Patients typically perceive spoken words as “meaningless noise,” “jabbering,” or an unrecognizable “foreign language” (8, 11, 12). Crucially, the recognition of environmental sounds and music is generally preserved, serving as a key diagnostic differentiator (8).

The pathognomonic profile of PWD includes significant impairments in auditory comprehension, repetition, and writing to dictation (1, 2, 5). In contrast, internal language remains undisturbed, and spontaneous speech production, reading comprehension, and spontaneous writing are typically spared (2, 4, 6, 7). Nevertheless, truly “pure” cases are exceptionally rare; most patients show accompanying expressive language impairments, such as logorrhea, phonemic paraphasias, or errors in naming and writing, and PWD is commonly diagnosed even when these contaminating symptoms are present (2, 4, 8, 10). While PWD is most commonly associated with stroke, it has also been described in relation to neurodegenerative diseases, infections, and seizures (4, 5, 10).

The incidence and prevalence of late-onset epilepsy (LOE) are increasing significantly as the global population ages (13–15). In individuals over 80 years of age, the annual incidence of epilepsy rises to more than 150 per 100,000 individuals (13, 14). LOE presents unique diagnostic challenges because seizure semiology in elderly patients often lacks classic motor features observed in younger populations (13, 15). Instead, seizures are primarily characterized by impairment of awareness, disturbed cognition, and acute confusion, which frequently mimic dementia, delirium, or psychiatric disorders (13, 15). Furthermore, up to 45% of elderly patients with new-onset seizures initially present with status epilepticus (SE), and the incidence of SE in the elderly is approximately twice that observed in younger adults (13, 15). While new-onset epilepsy in the elderly includes presentations that are difficult to recognize, such as non-convulsive status epilepticus (NCSE), even in cases involving overt convulsions—as seen in the present patient—subsequent higher brain dysfunction, specifically PWD, might be overlooked and underdiagnosed/underreported.

In this report, we describe a senile case of PWD related to LOE, contributing to the clinical understanding of this rare condition in the elderly.

## Case presentation

A 61-year-old right-handed man had a medical history of diabetes and hyperlipidemia. Six months prior to the current admission, he had experienced an episode similar to the present event. He was admitted to another hospital at that time, where he was diagnosed with a stroke and treated with antiplatelet therapy and lacosamide, followed by two months of rehabilitation. During this period, he was also diagnosed with mild cognitive impairment (MCI).

On the day of admission, the patient presented with generalized convulsive SE.

**Figure 1:**
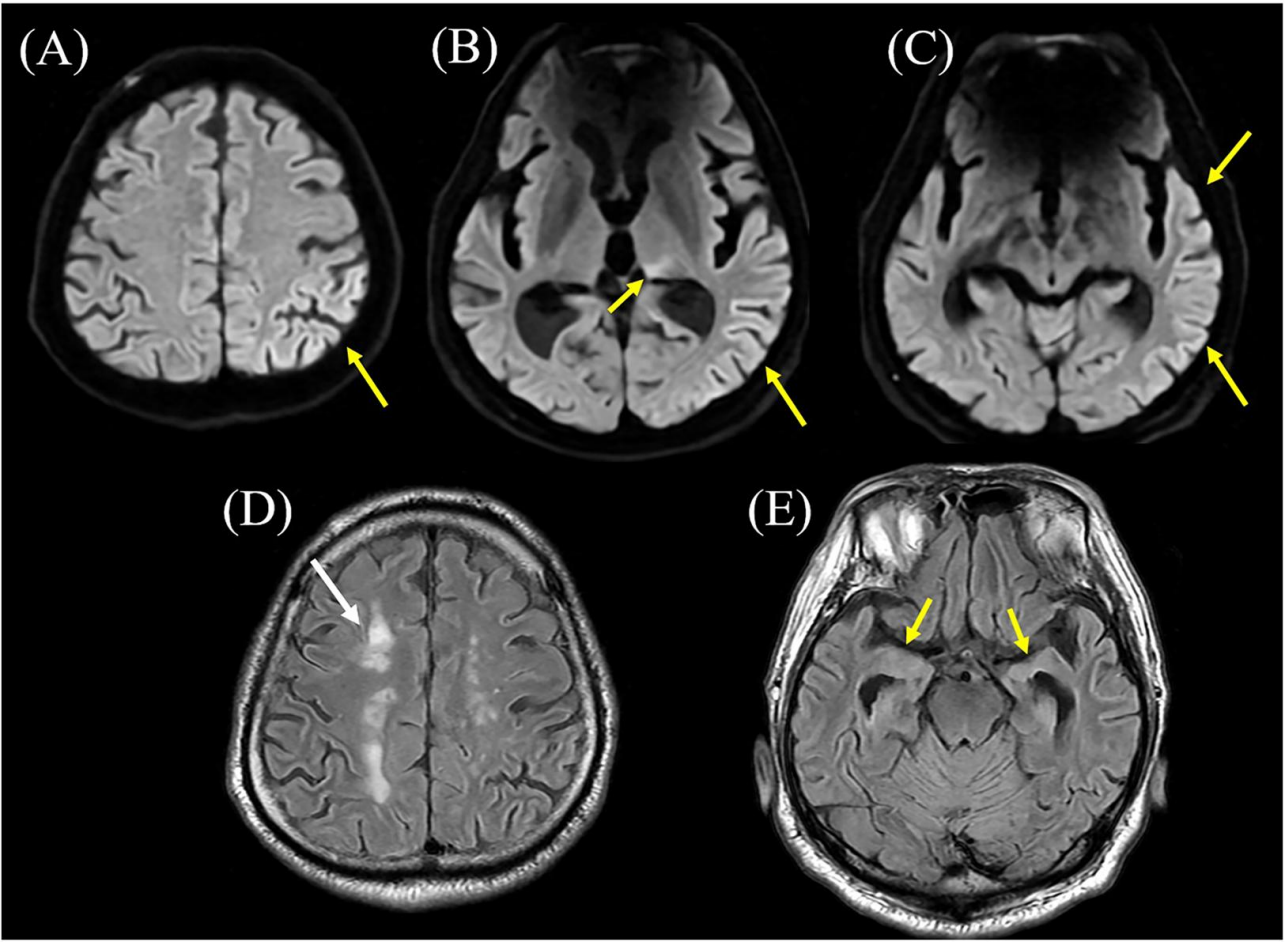
Brain magnetic resonance imaging (MRI) result. **(A–C)** Diffusion-weighted MRI shows hyperintensity in the left temporal and parietal lobes involving the thalamic pulvinar (arrows). These findings were resolved on follow-up imaging on day 16 (data not shown). **(D,E)** T2-FLAIR MRI reveals deep white matter lesions in the watershed region (white arrow) and atrophy of the medial temporal lobes, particularly the hippocampus (yellow arrows).

**Figure 2:**
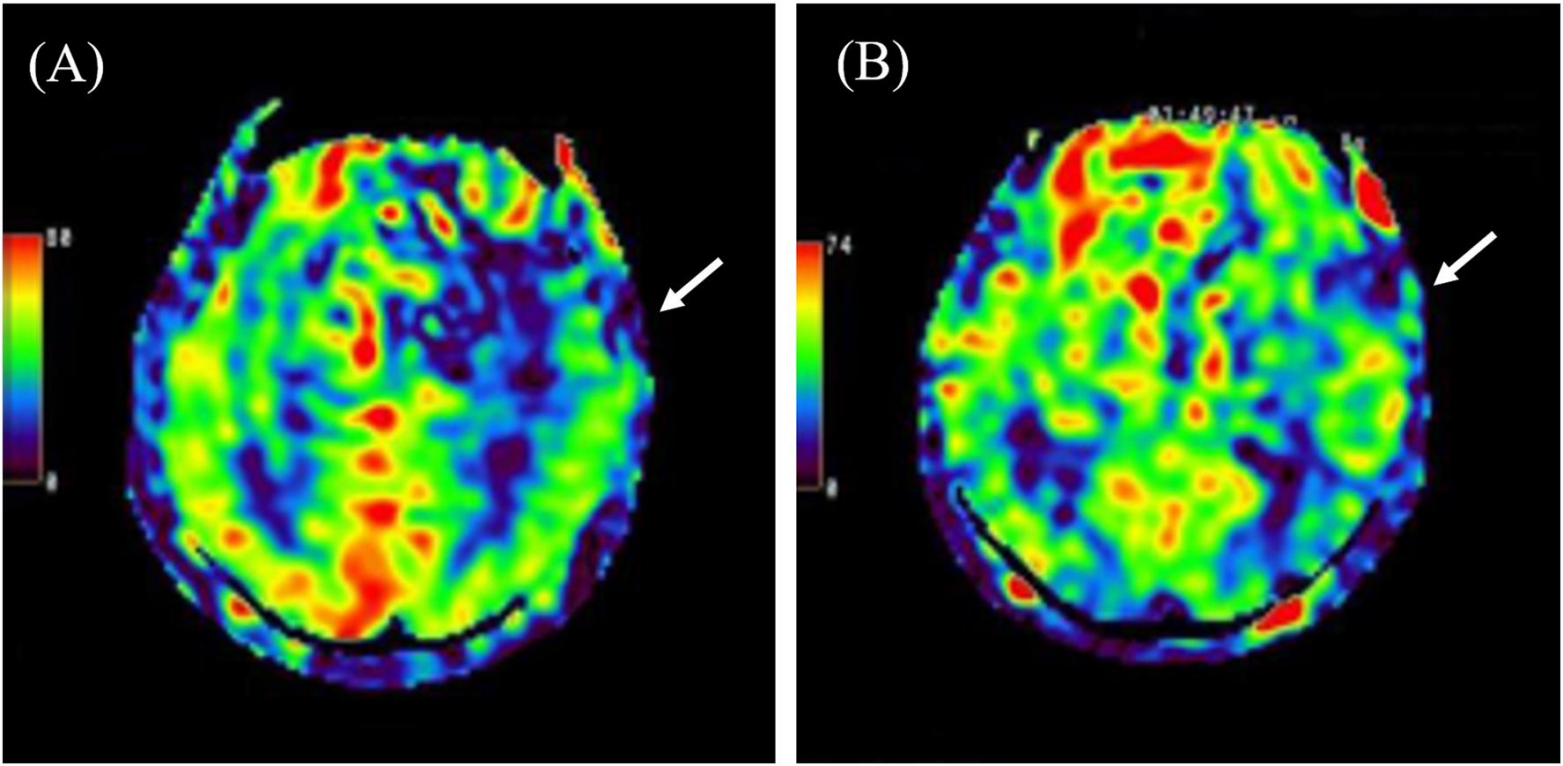
Arterial spin labeling (ASL) findings on admission and day 16. ASL demonstrated hypoperfusion in the left temporal lobe on admission **(A)**, which showed suggestive improvement on follow-up imaging performed on day 16 **(B)**.

The event began immediately after a subjective seizure aura described by the patient as “feeling strange” (an indefinable psychic or epigastric sensation). He initially experienced focal motor activity starting in the right upper limb, which rapidly evolved into a generalized tonic-clonic seizure. Immediately upon arrival, 5 mg of diazepam was administered intravenously, which successfully aborted the convulsive activity that had persisted for over 30 min. On admission, vital signs showed a body temperature of 37.7 °C, heart rate of 109 bpm, and blood pressure of 212/104 mmHg. The Glasgow Coma Scale score was E1V1M3. On initial clinical examination, prominent lateralizing features were observed, including right conjugate gaze deviation and head rotation to the right. While neurological findings could not be thoroughly assessed due to impaired consciousness, there was no evident gross paralysis or sensory deficit. Brain magnetic resonance imaging (MRI) showed hyperintensity involving the left temporal and parietal lobes and thalamic pulvinar on diffusion-weighted imaging (DWI) (Figure 1). Arterial spin labeling (ASL) demonstrated focal hypoperfusion in the left temporal lobe (Figure 2A), which showed suggestive improvement on follow-up imaging performed on day 16 (Figure 2B). Additionally, hippocampal and parietal atrophy were revealed. Cerebrospinal fluid (CSF) analysis revealed no pleocytosis, elevated protein (65.7 mg/dL) and glucose (100 mg/dL; concurrent blood glucose, 166 mg/dL) levels, and negative PCR results for herpes simplex virus and varicella-zoster virus. Whole-body computed tomography (CT) scan disclosed no tumorous lesions. Although electroencephalography (EEG) did not reveal significant epileptiform discharges, his clinical presentation and MRI findings suggested epilepsy; therefore, levetiracetam was administered. Additionally, because encephalitis could not be excluded, methylprednisolone therapy was also initiated.

**Figure 3:**
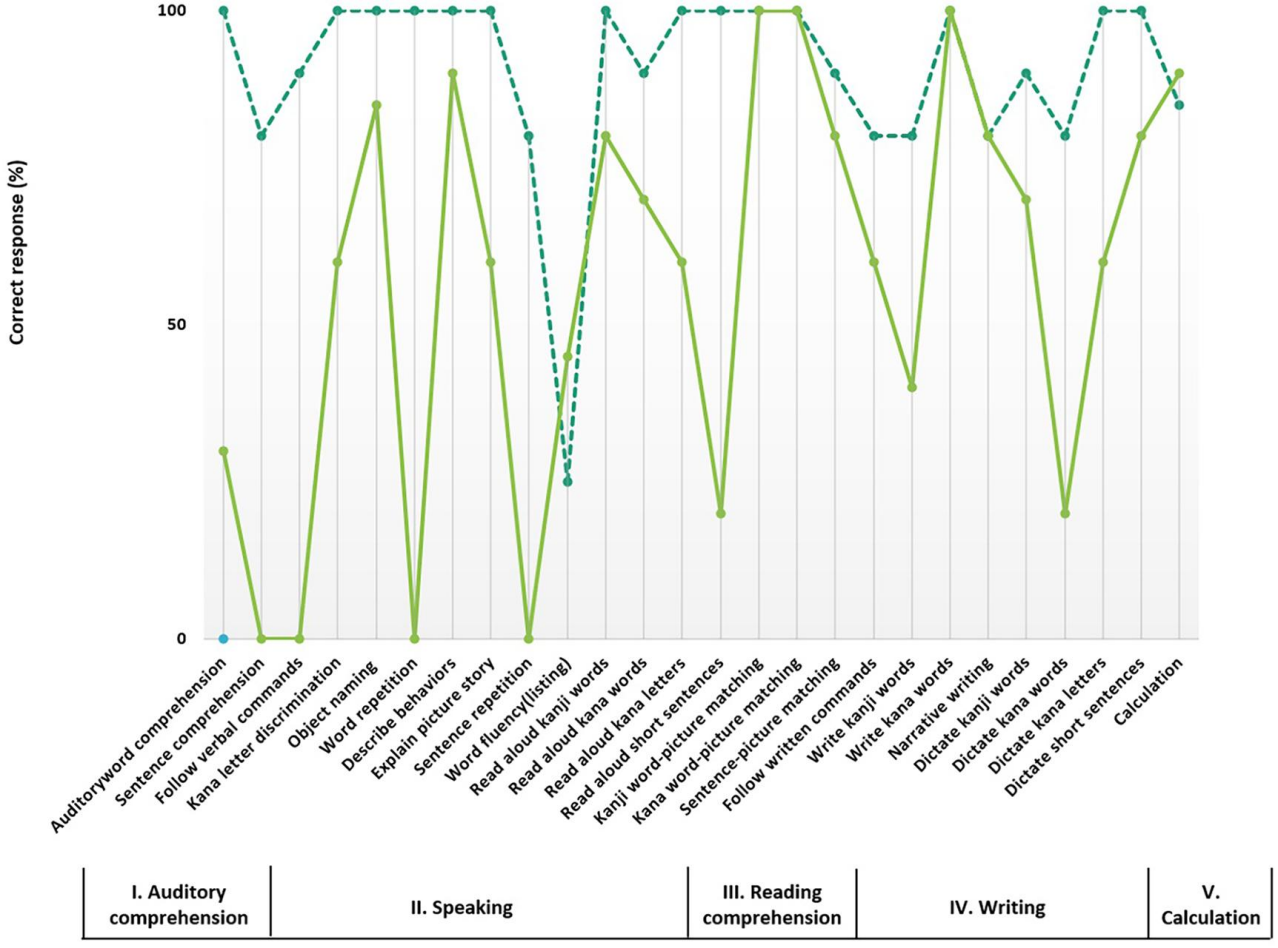
Profile of the Standard Language Test of Aphasia (SLTA). The results of SLTA on day 9 and 19. Solid line; score on day 9. Broken line; score on day 19. The Standard Language Test of Aphasia (SLTA), developed in 1975, is Japan's most reliable tool for classifying and assessing the severity of aphasia in Japanese speakers (12). It consists of 26 subtests that evaluate auditory comprehension, speaking, Reading comprehension, writing, and calculation (12). A previous study regarding aphasia by putaminal hemorrhage has demonstrated the effectiveness of treatment by comparing SLTA results before and after intervention (16). The SLTA results on day 9 showed impairments in severe auditory comprehension, word and sentence repetition, word fluency, Reading aloud short sentences, writing kanji (Chinese characters), and dictating kana (Japanese syllabary) characters, all below 50% performance. On day 19, a second SLTA was conducted, demonstrating significant improvement across nearly all subtests, except for word fluency.

The patient exhibited a state of delirium until day 3. His level of consciousness began to improve around day four; however, he demonstrated difficulty in responding to verbal commands. He reacted consistently to auditory stimuli such as finger scraping sounds, indicating preserved hearing function. Written language comprehension was intact, as evidenced by his ability to follow instructions presented on a whiteboard. Although his speech was fluent and grammatically correct, he exhibited a tendency toward perseveration. Neuropsychological assessments were conducted gradually as the patient's level of consciousness improved. Screening measures of global cognitive function were relatively preserved, with a Hasegawa Dementia Scale–Revised (HDS-R) score of 25 and a Mini-Mental State Examination (MMSE) score of 27. In contrast, the Frontal Assessment Battery (FAB) score was 8 out of 18, indicating marked executive dysfunction. On the Trail Making Test–Japanese version (TMT-J), the patient required 125 s to complete Part A and 115 s for Part B, suggesting reduced processing speed and impaired attentional control. Performance on the Kohs Block Design Test yielded an estimated IQ of 63, consistent with deficits in visuospatial and constructional abilities. Additionally, the patient scored 26 out of 36 on the Raven's Colored Progressive Matrices, reflecting diminished nonverbal reasoning and abstract thinking. On the Standard Language Test of Aphasia (SLTA), developed in 1975 and recognized as Japan's most reliable tool for classifying and assessing the severity of aphasia in Japanese speakers (12), the patient scored below 50% on subtests assessing auditory comprehension, repetition of words and sentences, word fluency, oral reading of short passages, and writing of kanji characters (Figure 3). The decline in “reading aloud short sentences” observed on day 9 may not be part of the core PWD symptoms. Instead, these might have reflected the extensive involvement of the left temporoparietal network as seen on MRI, combined with post-ictal fatigue following the prolonged status epilepticus, which can temporarily dampen high-level cognitive performance. Pure-tone audiometry revealed normal hearing thresholds, and his ability to match nonverbal sounds with corresponding pictures was excellent, with a score of 7 out of 8. Despite impaired oral reading, a diagnosis of PWD was made based on preserved peripheral hearing and environmental sound discrimination. Notably, his ability to match nonverbal sounds with corresponding pictures was excellent, with a score of 7 out of 8. His PWD persisted for several days but began to gradually improve around day 15. On the SLTA administered on day 19, a marked improvement in his language profile was observed (Figure 3). By day 20, his language skills had improved significantly, and he was able to converse. Although attentional dysfunction and memory decline remained, he was discharged from our hospital on day 38.

## Discussion

We reported a rare case of epilepsy-related PWD in an elderly patient. Although no clear ictal activity was observed on EEG, he experienced recurrent seizures, and brain MRI demonstrated hyperintensities in the left temporoparietal lobe as well as in the thalamic pulvinar, findings consistent with a post-ictal state. ASL findings were unique; while hyperperfusion is often observed during epileptic seizures, in this case, hypoperfusion was noted in the left temporal lobe, corresponding to hyperintensity on diffusion-weighted imaging (DWI). During the ictal phase or ongoing seizures, affected cortical or subcortical regions typically show hyperperfusion on ASL due to increased metabolic demand and neuronal activity (17–19). This reflects the increased cerebral blood flow required to meet the elevated oxygen and energy consumption. In contrast, early postictal or prolonged seizure stages may show relative hypoperfusion, possibly due to transient metabolic exhaustion, neuronal dysfunction, or seizure-induced vascular dysregulation (17, 18). This can manifest as reduced cerebral blood flow and thus decreased ASL signal in the same area. Therefore, the focal hypoperfusion observed in the left temporal lobe, corresponding to the area of DWI hyperintensity, was interpreted as consistent with the early postictal phase. However, it is important to acknowledge the limitations in the quantitative reliability of ASL for assessing subtle perfusion changes. Rather than serving as definitive evidence of hemodynamic shifts, these findings should be considered suggestive observations that correlate with the patient's clinical course of language recovery and the resolution of seizure activity. However, CSF biomarkers associated with Alzheimer's disease (AD) were not assessed, precluding a definitive conclusion regarding the contribution of neurodegenerative pathology—particularly AD—to the observed seizures. The possibility of symptomatic epilepsy due to encephalitis remained but was deemed unlikely, as the patient had experienced an epileptic seizure six months earlier and CSF findings revealed no pleocytosis and negative PCR results. Nonetheless, autoimmune encephalitis cannot be entirely ruled out.

PWD is typically associated with two primary lesion patterns: bilateral temporal lobe damage or unilateral left-hemisphere disconnection (4, 6, 10). Bilateral involvement commonly affects the superior temporal gyrus (STG), Heschl's gyrus, and the planum temporale, involving either the cortical gray matter or subcortical white matter (4, 6). Existing evidence underscores that early speech perception relies on bilateral auditory processing, explaining why many patients only develop persistent PWD following a second, contralateral stroke after an initial, often unnoticed, unilateral event (4, 6). Alternatively, PWD can manifest as a unilateral disconnection syndrome (4, 10). Historically, this mechanism has been attributed to a strategically located lesion in the deep white matter near the posterior part of the lateral ventricle, which disrupts two critical pathways: the ipsilateral auditory radiation and the commissural (transcallosal) fibers originating from the right hemisphere (4, 8). The simultaneous interruption of these pathways effectively results in the functional deafferentation of Wernicke's area (4, 8). In this model, even if the right auditory cortex remains perfectly intact and capable of processing acoustic signals, the resulting linguistic information cannot reach the language-dominant left hemisphere because the “bridge” provided by the transcallosal fibers is severed (8).

A critical question is how a primarily cortical phenomenon like epilepsy results in a clinical picture typically associated with white matter disconnection syndrome. While classic PWD often stems from permanent structural damage to the left auditory radiations and transcallosal fibers, recent evidence suggests that complete anatomical destruction is not always a prerequisite for the syndrome (4, 10). Maffei et al. proposed that functional disconnection can result from combined partial cortical and subcortical damage, where the remaining neural structures fail to process verbal input due to asymmetric or asynchronous signals (4).

In the context of epilepsy, although seizures are cortical events, the postictal state can induce transient metabolic exhaustion and neuronal dysfunction not only in the affected cortex but also within associated subcortical networks. Shijo et al. reported an adult case of PWD where focal seizure activity in the left superior temporal gyrus led to an immediate but reversible language deficit, supporting the idea that localized cortical dysfunction alone can functionally isolate Wernicke's area from auditory linguistic input (20). In our patient, the intense focal seizure activity in the left temporoparietal region—as evidenced by the initial MRI findings—might have caused localized metabolic depletion within the underlying white matter, encompassing these critical tracts. This exhaustion might have functionally inactivated the ipsilateral auditory radiation and the transcallosal fibers, effectively resulting in a “transient functional deafferentation” of Wernicke's area. This explains why our patient presented with the classic features of a unilateral disconnection syndrome despite the absence of a permanent anatomical lesion. The remarkable recovery of language function within 20 days coincides with the resolution of this metabolic exhaustion, distinguishing this functional isolation from the permanent structural destruction typically seen in vascular PWD.

Although the diagnostic hallmark of PWD is a selective deficit in auditory processing, truly “pure” cases are exceptionally rare in clinical practice (4). Most patients exhibit various accompanying expressive language impairments, such as logorrhea, phonemic paraphasias, or naming and writing errors, and PWD is often diagnosed even when these “contaminating” symptoms are present (2, 4, 8, 10). In our patient, while auditory comprehension was profoundly impaired, dictation scores were relatively preserved. This dissociation, though seemingly confusing, has been observed in other cases where phonological transcoding paths remain partially operational despite a severe breakdown in mapping auditory inputs to lexical-semantic representations (4, 5, 7). For example, Salemme et al. reported a case where a striking disproportion between spoken comprehension and written tasks was evident, suggesting that residual “islands” of functional processing can exist within the spectrum of PWD (7). Furthermore, as noted by Maffei et al., PWD frequently evolves from or is accompanied by broader language deficits during the acute phase (4), suggesting that our patient's SLTA profile reflects a focal clinical peak within a wider landscape of temporoparietal dysfunction rather than a primary dissolution of Wernicke's area itself.

Epilepsy has been identified as a potential cause of PWD in pediatric populations, particularly in relation to Landau-Kleffner syndrome (LKS), a rare form of epileptic encephalopathy that affects otherwise healthy children, typically between ages 3 and 8 (10–25). The defining feature of LKS is a severe impairment in speech comprehension, often referred to as verbal auditory agnosia or “word deafness,” which is accompanied by nearly continuous epileptiform discharges on EEG during slow-wave sleep (21, 23–26). While approximately 70%–80% of affected children experience clinical seizures and behavioral issues are common, the epileptic activity itself is generally regarded as the primary factor underlying language deterioration (21, 23–27). Beyond pediatric cases, reports of PWD in adults are infrequent but do exist. One such case involves a 31-year-old right-handed man who developed PWD following a localized epileptic seizure; his diagnosis was confirmed through EEG and neuroimaging, and he experienced immediate recovery after administration of midazolam, representing a rare adult instance of seizure-induced PWD (20). Another reported case concerns a 49-year-old man who developed PWD after suffering a grand mal seizure; he was unable to comprehend spoken language but maintained normal speech, reading, and writing abilities. Continuous seizures and EEG findings indicated ongoing seizure activity in the left temporal lobe (1). Additionally, a 52-year-old man presenting with PWD and simple focal seizures exhibited EEG abnormalities characterized by sharp spikes in the left temporal region; his condition was diagnosed as paraneoplastic encephalitis associated with small cell lung cancer, and he was treated with gabapentin (28). A 24-year-old woman developed PWD within a week after experiencing two seizures caused by herpes simplex virus type 1 encephalitis; MRI scans revealed bilateral brain lesions, predominantly involving the left superior and transverse temporal gyri, and she received anticonvulsant therapy (29). In summary, PWD in adult epilepsy cases tends to involve lesions in the left temporal lobe, aligning with the current understanding that the left hemisphere predominantly manages language processing. Damage to this region—particularly disruption of the connection between the primary auditory cortex and language areas such as Wernicke's area—can lead to the development of PWD.

Our case is particularly noteworthy as temporally PWD developed in the context of epilepsy in the senile. The patient, after admission, was in a coma with presumed post-epileptic encephalopathy. Following his recovery from this state, he exhibited a marked dissociation between auditory and visual modalities: he was unable to comprehend spoken language but could follow written commands. The SLTA results led us to suspect that his speech symptoms were related to PWD; however, it should be noted that the SLTA is not a standardized tool for diagnosing PWD. The SLTA results showed impairments in severe auditory comprehension, word and sentence repetition, reading aloud short sentences, writing kanji (Chinese characters), and dic-tating kana (Japanese syllabary) characters, all below 50% performance. Peripheral hearing and environmental sound discrimination were preserved. Based on the core symptoms of PWD—severe deficits in auditory comprehension, repetition and writing to dictation, as well as preserved understanding of non-speech sounds—the patient had a strong indication of PWD; however, the additional impairments in reading aloud suggest that this is not a “pure” case. Given that PWD often involves mild expressive language impairments and many cases are diagnosed and managed as such even with additional symptoms (2, 4), we believe our case corresponds to PWD. While elderly patients with epilepsy frequently present with dementia-like symptoms, including unresponsiveness to verbal commands (30), there are few reports specifically addressing the potential inclusion of PWD within these presentations. One reason for this may be that, when caused by epileptic seizures, these symptoms are reversible (as observed in this case, with recovery within 20 days), leading to the possibility that transient PWD often goes unnoticed and remains underdiagnosed and underreported. Therefore, our findings underscore the critical importance of recognizing PWD as a potential, albeit rare, consequence of epilepsy in the elderly and warrant further investigation into its underlying mechanisms.

This study has several limitations that warrant consideration. First, standard scalp EEG failed to detect definitive epileptiform discharges. Although significant epileptiform abnormalities are frequently subtle or absent on EEG in elderly patients or during the postictal phase (31), the use of prolonged EEG monitoring or magnetoencephalography might have provided stronger electrophysiological evidence of the suspected seizure activity. Second, the precise etiology of the patient's epilepsy remains unconfirmed. While MRI showed chronic ischemic changes in the deep white matter, no acute cortical lesions, such as cerebral infarction or hemorrhage, were identified. The presence of prominent hippocampal atrophy raised suspicion of underlying AD; however, AD-specific biomarkers in the cerebrospinal fluid were not assessed. Given the increasing availability of such tests, their inclusion would have allowed for a more definitive evaluation of a possible neurodegenerative contribution to seizures. Furthermore, although the clinical course and recurrent nature of the events made acute infectious, autoimmune, or paraneoplastic encephalitis less likely, these diagnoses could not be entirely ruled out. Our screening was limited to HSV and VZV; a more comprehensive antibody panel would have allowed for a more thorough diagnostic evaluation.

## Conclusion

This report describes a rare case of epilepsy-related PWD in an elderly patient. Elderly patients with epilepsy frequently exhibit dementia-like symptoms, such as unresponsiveness to verbal commands, and our findings suggest that PWD may represent a specific, often overlooked manifestation of these clinical presentations. Because epileptic symptoms and their postictal effects are typically transient and reversible—as demonstrated by this patient's significant recovery within 20 days—such PWD cases likely go unrecognized, are misdiagnosed as acute cognitive decline, and remain underreported. Therefore, further clinical documentation is essential to clarify that PWD can represent a focal manifestation of late-onset epilepsy.

## Data Availability

The original contributions presented in the study are included in the article/Supplementary Material, further inquiries can be directed to the corresponding author.
